# Analysis of Trigger Factors in Episodic Migraineurs Using a Smartphone Headache Diary Applications

**DOI:** 10.1371/journal.pone.0149577

**Published:** 2016-02-22

**Authors:** Jeong-Wook Park, Min Kyung Chu, Jae-Moon Kim, Sang-Gue Park, Soo-Jin Cho

**Affiliations:** 1 Department of Neurology, the Catholic University of Korea College of Medicine, Seoul, Korea; 2 Department of Neurology, Sacred Heart Hospital, Hallym University College of Medicine, Anyang, Korea; 3 Department of Neurology, Chungnam National University College of Medicine, Daejeon, Korea; 4 Department of Applied Statistics, Chung-Ang University, Seoul, Korea; 5 Department of Neurology, Dongtan Sacred Heart Hospital, Hallym University College of Medicine, Hwaseong, Korea; Taipei Veterans General Hospital, TAIWAN

## Abstract

**Background:**

Various stimuli can trigger migraines in susceptible individuals. We examined migraine trigger factors by using a smartphone headache diary application.

**Method:**

Episodic migraineurs who agreed to participate in our study downloaded smartphone headache diary application, which was designed to capture the details regarding headache trigger factors and characteristics for 3 months. The participants were asked to access the smartphone headache diary application daily and to confirm the presence of a headache and input the types of trigger factors.

**Results:**

Sixty-two participants kept diary entries until the end of the study. The diary data for 4,579 days were analyzed. In this data set, 1,099 headache days (336 migraines, 763 non-migraine headaches) were recorded; of these, 772 headache events had with trigger factors, and 327 events did not have trigger factors. The common trigger factors that were present on headache days included stress, fatigue, sleep deprivation, hormonal changes, and weather changes. The likelihood of a headache trigger was 57.7% for stress, 55.1% for sleep deprivation, 48.5% for fatigue, and 46.5% for any trigger. The headaches with trigger factors were associated with greater pain intensity (p<0.001), headache-related disability (p<0.001), abortive medication use (p = 0.02), and the proportion of migraine (p < 0.001), relative to those without trigger factors. Traveling (odd ratios [OR]: 6.4), hormonal changes (OR: 3.5), noise (OR: 2.8), alcohol (OR: 2.5), overeating (OR: 2.4), and stress (OR:1.8) were significantly associated with migraines compared to non-migraine headaches. The headaches that were associated with hormonal changes or noise were more often migraines, regardless of the preventive medication. The headaches due to stress, overeating, alcohol, and traveling were more often migraines without preventive medication, but it was not evident with preventive medication.

**Conclusion:**

Smartphone headache diary application is an effective tool to assess migraine trigger factors. The headaches with trigger factors had greater severity or migraine features. The type of triggers and the presence of preventive medication influenced the headache characteristics; hence, an investigation of trigger factors would be helpful in understanding migraine occurrences.

## Introduction

Migraines are characterized by recurrent headaches and a hypersensitivity to sensory stimuli [1]. Various external and internal stimuli can lead to migraine events in susceptible individuals, which can be considered as migraine triggers or precipitants [2, 3]. The reported migraine triggers include stress, sleep, fatigue, fasting, physical exercise, hormonal changes, weather, sunlight, alcohol, and various sensory stimuli [4–9]. Most studies that examined migraine trigger factors were based on participant reports. These trigger factors are found in 73–80% of migraineurs [10, 11].

Most migraineurs encounter their migraine triggers daily without suffering a migraine attack, and only some migraineurs experience a migraine attack provoked by a trigger factor [4]. To avoid general exposure to migraine triggers, a process to identify personal headache triggers in real life may be useful for migraine sufferers. Moreover, the estimation of the degree of each trigger factor’s contribution to a headache occurrence is meaningful. Schurks et al. recently proposed that triggering or precipitating factors were important for the characterization of migraine phenotypes, despite being excluded from the current diagnostic criteria for a headache disorder [12]. Diary studies using advanced statistical modeling techniques may be required for the reliable identification of one’s migraine trigger factors [13, 14]. In a paper diary study using a Cox regression analysis, hormonal changes exerted the most prominent influence, increasing the likelihood of a headache occurrence [15].

While most patients have reported one or more triggers, the major limitation to accepting these triggers as precipitating factors resides in the reliability of the patient’s migraine trigger recall and selection [3, 10]. In addition, the causal relationships between triggers and migraines are currently uncertain.

Traditionally, the paper diary has been used to investigate the association between a trigger exposure and a migraine attack. Electronic diaries may be superior to paper diaries in that they offer advantages, such as a reduction of recall bias, easy accessibility for physicians and patients, and improved compliance [16–19]. Recently, advances in information technology have resulted in the development of electronic headache diaries using hand-held devices, such as smartphones. However, only a few headache diary studies using smartphones or handheld devices have evaluated the association between trigger exposures and migraine attacks [17, 20]. Computer-based electronic diaries require special equipment, such that real-time data collection can be difficult [16, 20]. In order to overcome the limitations associated with an internet-based online diary, we created the Smartphone Headache Diary Application (SHD), which is easy to use and accessible, in order to enable patients to record data on a near real-time basis [21–23].

Therefore, in this study, we investigated the frequencies and impacts of triggers in episodic migraine patients using our SHD.

## Methods

### Participants and baseline survey

The participants who met the inclusion criteria were recruited between September 2014 and January 2015. This study was conducted at the neurology outpatient clinics of university hospitals.

The following inclusion criteria were applied: 1) age between 19 and 55 years and migraines with or without auras, as defined by the International Headache Society Criteria for Migraine (ICHD-3 beta) [24]; 2) an of 2–14 headache days per month; 3) stable headache characteristics for at least 1 year prior to study entry; and 4) the possession of a personal platform smartphone that was capable of operating the SHD.

The following exclusion criteria were applied: 1) headaches attributed to secondary causes; and 2) inability to complete questionnaires, to use our specially designed SHD, or to comply with the SHD usage requirements.

For the baseline survey, the participants were asked to choose their potential triggers on the basis of their previous experiences from a list of 18 trigger factors. Those factors were selected on the basis of the results of previous studies about migraine trigger factors, and included stress, excessive sleep, sleep deprivation, exercise, fatigue, hormonal changes, emotional changes, weather changes, sunlight, noise, odors, fasting, overeating, caffeine, smoking, alcohol, cheese/chocolate, and traveling [2, 5, 6, 8, 9]. The participants were also asked to complete the Hospital Anxiety and Depression Scale to determine their anxiety and depression levels [25]. They also completed the Korean versions of the Migraine Disability Assessment Scale (MIDAS) and the Headache Impact Test-6 to determine the impact of their migraines on their daily functioning [26, 27].

The ethical approval for the study was granted by the Dongtan Sacred Heart Hospital Institutional Review Board/Ethic Committee (IRB approval number: 2014–132) and Uijeongbu St.Mary’s Hospital, the Catholic University of Korea College of Medicine Institutional Review Board/Ethic Committee (IRB approval number: UC14OIM10085). The participants received an explanation of the study’s aims and procedures and provided written informed consent.

### SHD development and contents

Two registered nurses, a project coordinator, a web-support project manager, and three headache specialists developed the SHD together.

The SHD included systematic instructions for its use during the study. The patients’ recorded SHD data were available to them after their enrollment in the study.

A series of reports, which included each participant’s missing diary days, was automatically uploaded. These reports allowed researchers to track each participant’s status and alert the participants if increased compliance was needed. The SHD was programmed to check the participants’ incidence of headache and the potential triggers on a daily basis. In addition to the potential trigger factors identified at the initial assessment, the participants were able to input details concerning other headache triggers.

### Data collection using the SHD

Subsequent to SHD installation, a registration number and password were provided to each participant to maintain security. The participants could upload information into the diary simply by touching the screen. They were asked to complete the SHD on a daily basis for 3 months. A short message was sent every two weeks to remind the participants to enter information.

Every day, regardless of the presence of a headache, the participants were asked to touch the SHD icon shown in their smartphone. After doing so, they could log into the SHD. The first screen asked the patient whether he/she had a headache. If the patients noted that there was no headache, then it was automatically recorded as a ‘no headache day’. However, if there was any form of a headache present, he/she was asked to record the headache characteristics their headache (e.g., headache intensity, duration, and the presence of photophobia or phonophobia), any headache self-treatment, and headache-related functional disability (e.g., MIDAS, if any). Finally, the patients were asked to select the triggers from the list of 18 trigger factors presented during the same day. The triggers present during the 1–3 days preceding the headache were not considered or selected. In addition, the patients were asked to record those trigger factors daily, regardless of the presence of a headache.

The participants were interviewed by the researchers and received a personal summary of their daily records for the preceding 3 months at the study’s completion.

### Data analysis

We analyzed the effect of trigger factor exposure on the headache occurrence using the daily records from the participants’ diary entries. The frequency for each trigger factor was acquired by calculating the number of headache days with certain trigger factors divided by the total number of headache days. There were many terms for the occurrence of a headache, such as intensity or probability; we chose the likelihood of a headache [3, 19]. Likelihood of a headache during the presence of each trigger factor was obtained with the following equation:

$$\text{Frequency}=\frac{\text{the number of headache days with certain trigger factors }}{\text{total number of headache days}}\times 100$$

$$\text{Likelihood }=\frac{\text{the number of headache days with certain trigger factor}}{\text{the number of days with presence of the same trigger factor}}\times 100$$

Each headache was classified as a migraine or non-migraine headache, according to the diagnostic criteria B–D of item 1.1 of migraine without aura in the ICHD-3 beta.

The categorical variables were presented as percentages, and the continuous variables were summarized using descriptive statistics, such as the means and standard deviations. The clinical variables for the headache were compared according to the presence or absence of trigger factors, using t-tests for continuous variables, and a chi-square test or Fisher’s exact test for the frequency variables.

The trigger factor frequency was compared between the migraine and non-migraine headaches by using a chi-square test or Fisher’s exact test. The associations of the 18 trigger factors and migraine were examined using a stepwise multiple logistic regression analysis with 153 possible combinations of trigger factors. A variable must have had a p value of less than 0.15 to be entered into the regression model. SAS statistical software (SAS version 9.3, SAS Institute, Inc., Cary, NC) was used for all analyses. The statistical significance was set at p < 0.05.

## Results

### Demographic characteristics

Initially, 113 patients were recruited from two centers. However, 30 patients withdrew before the end of the study; therefore, 83 patients finished the study. Of these, 62 patients kept a diary for at least 50% of the study period. We analyzed the headache diary data from 62 patients (S1 File). Sixty patients had a migraine without aura, and two had a migraine with aura. The participants’ mean age was 37.7 ± 8.6 years of age, with 83% of participants being women. The mean illness duration was 9.7 ± 8.2 years (Table 1).

**Table 1:** Demographic and headache characteristics of the participants.

**Age, years**	37.7±8.6
**Female**	82.3%
**Duration of illness, years**	9.7±8.2
**Pain intensity, VAS**	7.5±1.3
**Monthly Headache days**	6.4±5.1
**Headache duration, hours**	31.1±26.3
**Frequency of abortive treatment per month**	4.6±3.6
**Current prophylactic medication**	40.3%
**HIT-6**	62.4±9.7
**MIDAS**	22.0±24.5
**HADS-D/ HADS-A**	9.5±13.9 / 6.5±3.1

Mean ± standard deviation; VAS, visual analogue scale; HIT-6, Headache impact test-6; MIDAS, Migraine Disability Assessment Scale; HADS-D, Score of Hospital anxiety depression scale-depression part; HADS-A, Score of Hospital anxiety depression scale-anxiety part

The required recording time per day was 2.1 ± 1.2 (1–5) minutes. All patients preferred to use the smartphone diary rather than the paper diary, as assessed by a survey after the end of the study.

### Personal triggers estimated at the baseline survey and on the SHD

At the initial study session, the participants were asked to provide a retrospective estimate of the number of migraine triggers of which they were aware. Of the participants analyzed, 39 (62.9%) reported any trigger(s) and the median number of triggers was 3 (range: 0–11). The participants estimated that 64.5% of their headaches were related to triggers.

In total, 4,579 diary days were recorded from 62 patients, with an 86.3% recording rate during 85 ± 13.4 days. The median number of headaches per patient was 15 during the period (range: 4–60). The proportion of patients who reported any trigger(s) on the SHD was higher than that found in the baseline survey (80.6% vs. 62.9%, p = 0.002). The number of possible trigger(s) for each patient was higher on the SHD [median: 7 (range 0–17)] than that in the baseline survey [median: 3 (range 0–11)].

### Trigger frequencies and likelihood of headache in presence of the triggers on the whole SHD

**Fig 1:**
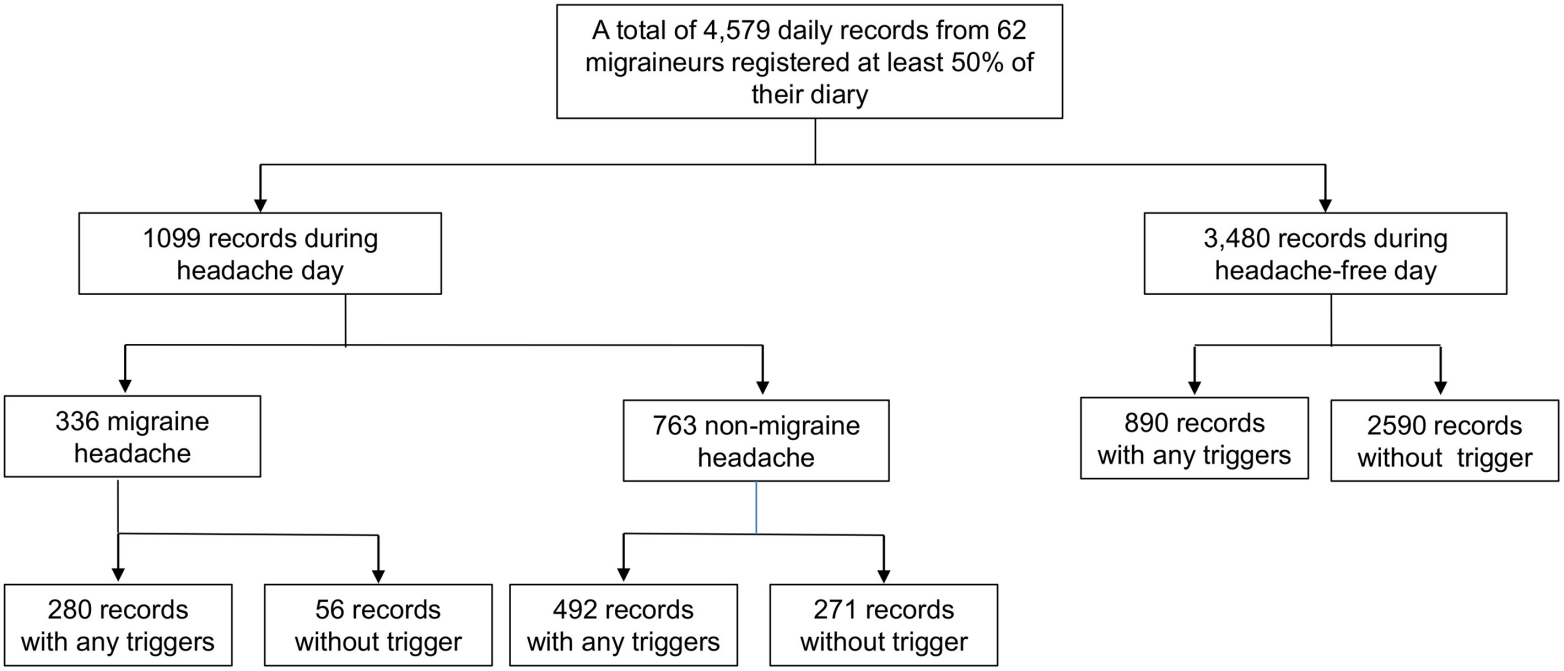
Flow Chart Depicting Subject Participation and the Number of Records. There were 722 headache days with certain triggers and 357 headache days without triggers. In total, 1662 days with a trigger and 2917 days without a trigger were recorded.

During the study period, 1,099 headache days were recorded (Fig 1). The median number of triggers on each recording headache day was 2 (range: 1–9); 80% of the headache diary listed 0–2 triggers (Table 2). The proportion of days with the presence of a trigger was 65.7% on the headache day and 25.6% on a day without a headache. There was no influence of the trigger number on the likelihood of a headache in the Chi-square analysis (S1 Table).

**Table 2:** The Number of Trigger of each Recording Day with and without Headache.

Number of triggers	0	1	2	3	4	5	6	7	8	9	Total
**Headache days**	327	305	247	139	56	18	3	2	1	1	1099
**No headache**	2587	351	267	182	76	14	1	0	1	1	3480
**Total**	2914	656	514	321	132	32	4	2	2	2	4579

**Fig 2:**
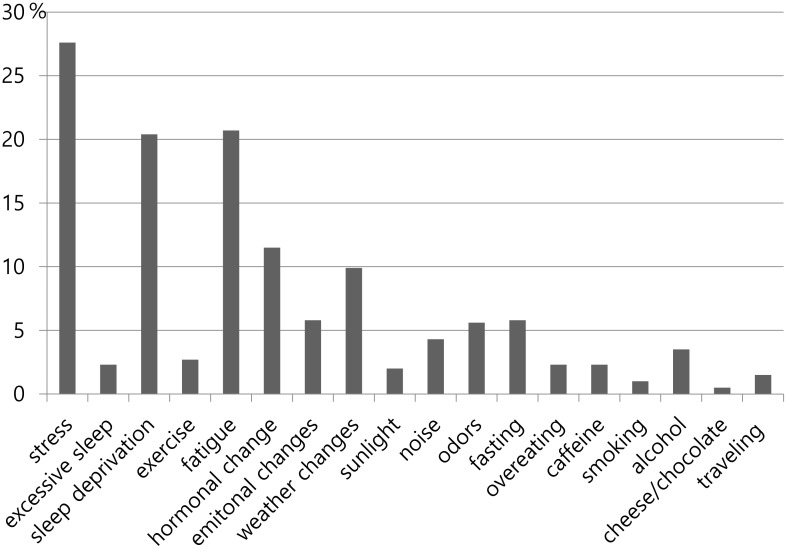
Distribution of each Trigger related to Headache according to Their Frequency.

When headaches were reported in the SHD, the common triggers were stress (27.6%), followed by fatigue (20.7%), sleep deprivation (20.4%), hormonal changes (11.5%), and weather changes (9.9%) (Fig 2).

Of the 1662 days that included a trigger, 772 day records (46.5%) were recorded as headache days. The following triggers were likely to trigger a headache: 78.6% for alcohol, 71.8% for odor, 68.8% for emotional change, 64.2% for hormonal changes, and 57.7% for stress, 55.1% for sleep deprivation, and 48.5% for fatigue.

### Analysis of headache characteristics among the 1099 headache day records on the SHD

Of the total number of headache days, 70.2% (772/1,099) were recorded as days that included triggers (Fig 1).

The headaches with triggers were related to a greater pain intensity (p < 0.001), the use of abortive treatment (p = 0.015), headache-related disability (p < 0.001), and the proportion of migraine (p < 0.001) than were those without triggers (Table 3). The headache duration did not differ between the two groups (p = 0.57).

**Table 3:** Comparison of the Headache Variables according to Trigger presence on a Smartphone Application-based Electronic Headache Diary.

Headache Variables	Headache not associated with Trigger (N = 327)	Headache associated with Trigger (N = 772)	P-value
Pain intensity, VAS	3.9±2.0	4.6±2.3	<0.001
Duration of headache, hours	7.7±5.7	8.0±5.8	0.57
Usage of abortive treatment	56.9%	64.8%	0.02
Disability associated with headache	33.6%	53.6%	<0.001
Proportion of migraines	17.1%	36.3%	<0.001

Mean ± standard deviation; VAS, visual analogue scale

Of the total number of headache days, 30.6% (336/1,099) of the headaches met the criteria for a migraine (Fig 1).

The presence of stress, sleep deprivation, hormonal changes, noise, odors, alcohol, fasting, overeating, cheese/chocolate consumption, and traveling were significantly more frequent in migraines relative to non-migraine headaches (Table 4). A stepwise multiple logistic regression analysis with the 18 trigger factors and the 153 possible combinations of the trigger factors revealed that traveling (odds ratio [OR]: 6.4, confidence interval [95% CI]::1.2–10.2), hormonal changes (OR: 3.5, 95% CI: 2.3–5.2), noise (OR: 2.8, 95% CI:1.4–4.9), alcohol (OR: 2.5, 95% CI:1.3–5.0), overeating (OR: 2.4, 95% CI:1.1–5.7), and stress (OR: 1.8, 95% CI:1.4–2.4) were significantly associated with migraines (Table 4). Two trigger combinations were selected during the stepwise logistic analysis. In situations with stress, but without hormonal changes, the OR did not change compared to the OR of stress alone. In situations with stress and hormonal changes, the migraine risk was not significant. The associations of noise and travel were similar (Table 4).

**Table 4:** Comparison of Triggers between Migraines and Non-migraine headaches on the Smartphone Application-based Electronic Headache Diary.

Triggers	NMH (N = 763)	Migraine (N = 336)	P-value*	Stepwise multiple logistic regression analysis	
				Odd ratio (95% confidence interval)	P-value
Stress	24.0%	36.0%	<0.001	1.8 (1.4–2.4)	<0.001
Excessive sleep	2.2%	2.4%	0.88	NA	
Sleep deprivation	18.6%	24.4%	0.03	NA	
Exercise	1.3%	1.5%	0.78	NA	
Fatigue	19.9%	22.3%	0.37	NA	
Hormonal changes	6.8%	18.5%	<0.001	3.5 (2.3–5.2)	<0.001
Emotional changes	5.1%	7.4%	0.13	NA	
Weather changes	10.8%	8.0%	0.17	NA	
Sunlight	2.1%	1.8%	0.73	NA	
Noise	2.6%	8.0%	<0.001	2.8 (1.4–4.9)	0.002
Odors	3.9%	9.2%	<0.001	NA	
Fasting	4.5%	8.9%	0.003	NA	
Overeating	1.3%	4.5%	0.001	2.4 (1.1–5.7)	0.009
Caffeine	2.2%	2.4%	0.88	NA	
Smoking	1.1%	0.6%	0.73	NA	
Alcohol	2.2%	6.3%	<0.001	2.5 (1.3–5.0)	0.009
Cheese/chocolate	0%	1.5%	0.003	NA	
Traveling	0.8%	3.3%	0.002	6.4 (1.2–10.2)	0.003
Stress*Hormonal changes = 0	24.0%	38.1%	<0.001	1.8 (1.3–2.5)	0.03
Stress*Hormonal changes = 1	31.9%	23.7%	0.31	0.7 (0.3–1.6)	
Noise*Travel = 0	2.4%	8.1%	<0.001	2.8 (1.4–5.4)	0.01
Noise*Travel = 1	42.9%	10.0%	0.12	0.1 (0.1–1.2)	

* Chi-square analysis

NMH, Non-migraine Headaches; NA, not available due lack of inclusion in the stepwise multiple regression analysis

The headaches with the presence of hormonal changes or noise were more likely to be migraines, regardless of any preventive medications. The headaches with the presence of stress, overeating, alcohol, and traveling were more often migraines without the use of preventive medication, but migraines were not evident with the use of preventive medication. The headaches with the presence of odors were more likely to be migraines only with preventive medication. The influences of the other trigger factors on the headache type were not different with the use of preventive medication (Table 5).

**Table 5:** Influence of Preventive Medication on the Headache Features with their Trigger Factors.

Triggers	without Preventive Medication (n = 579)			with Preventive Medication (n = 520)		
	NMH (n = 417)	Migraine (n = 162)	P-value	NMH (n = 346)	Migraine (n = 174)	P-value
Stress	25.7%	43.8%	<0.001	22.0%	28.7%	0.09
Excessive sleep	1.4%	1.9%	0.72	3.2%	2.9%	0.85
Sleep deprivation	16.6%	22.2%	0.12	21.1%	26.4%	0.85
Exercise	0.5%	1.2%	0.31	2.3%	1.7%	0.76
Fatigue	19.4%	22.8%	0.36	20.5%	21.8%	0.73
Hormonal changes	8.4%	20.4%	<0.001	4.9%	16.7%	<0.001
Emotional changes	6.0%	9.9%	0.10	4.1%	5.2%	0.56
Weather changes	10.8%	6.8%	0.14	10.7%	9.2%	0.59
Sunlight	0.7%	0.6%	1.00	3.8%	2.9%	0.60
Noise	2.2%	6.2%	0.02	3.2%	9.8%	0.002
Odors	1.9%	1.9%	1.00	6.4%	16.1%	<0.001
Fasting	4.6%	9.3%	0.03	4.3%	8.6%	0.05
Overeating	1.7%	7.4%	<0.001	0.9%	1.7%	0.41
Caffeine	2.2%	4.9%	0.10	2.3%	1.7%	0.41
Alcohol	3.1%	9.9%	<0.001	1.2%	2.9%	0.17
Traveling	0.2%	2.5%	0.02	1.5%	4.0%	0.12

Smoking and cheese/chocolate were not analyzed because more than one cell was zero. NMH, Non-migraine Headaches

## Discussion

The main findings of the current study were as follows: 1) the SHD was comprehensive in detecting the trigger factors of episodic migraineurs; 2) the frequent trigger factors on headache days were stress, fatigue, and sleep deprivation; the likelihood of a headache was 57.7% for stress, 55.1% for sleep deprivation, 48.5% for fatigue, and 46.5% for any trigger; 3) the headaches with trigger factors were more severe relative to those without trigger factors, 4) traveling, hormonal changes, noise, alcohol, overeating and stress increased the risk of migraines; and 5) hormonal changes and noise increased the risk of migraine regardless of preventive medication, whereas stress, overeating, alcohol, and traveling increased the risk of migraine in situations without preventive medication.

Our SHD was developed for clinical and research purposes. It has major advantages for researchers, including data entry by the participants, big data set collection, immediate and frequent data analysis, and no requirements for special diary equipment [22, 23]. For participants, SHD was easy to use. The summary report could be obtained from the clinician and the SHD after the end of the study. The SHD facilitated the research regarding migraine trigger factors, and has a great potential to enhance the communication between the physician and their patient [22, 28].

Among the 1099 headache days, 70.2% were related to trigger factors in this study. Compared to the baseline survey that was based on life experience, the actual frequency of each trigger factor was lower. The contributions of individual trigger factors were similar, and the proportion of patients who reported any trigger(s) was higher in the SHD for 3 months. The frequent trigger factors in this analysis were in line with previous studies [2, 3, 10, 29–30]. Several studies have suggested that stress is related to hormones, including cortisol and thyroid hormone, and may have a role in migraine pathogenesis [2, 29]. Fatigue was considered to be a migraine-specific trigger and may be related to sleep quality [31]. Sleep disturbances were 3–17 times more likely to be triggers for migraines, tension-type headaches, and chronic headaches in a population-based study. It has been suggested there is an overall pain-promoting effect of sleep deprivation [32]. Sleep deprivation was a predictor for severe headaches among chronic migraine patients, but was not associated with episodic migraines in this study [33]. Stress, fatigue, and sleep deprivation may be modifiable triggers for migraines, so proper attention to psychiatric disorders, sleep hygiene, and life habits is important in learning to cope with triggers [33–35]

The likelihood of a headache on a day with any trigger factor was 46.5%. Alcohol, odor, and emotional changes were not frequently associated with headaches, but the likelihood of a headache occurrence was more than 70% when the patients were exposed to those factors. Although the avoidance of all triggers is impractical in everyday life, the avoidance or pre-emptive approach for certain triggers may be reasonable [11].

The additional effects of triggers were not seen in the logistic analysis with 153 possible combinations of two triggers, although stress*menstruation and noise*travel were selected in the model. A previous study using natural triggers in migraine auras showed no additional effects of a flickering light and physical activity [4]. One study about odorant-triggered migraines showed the association of perfume odors within other factors, such as cleaning, cooking, beauty products, and foul odors [6]. The addictive effect of triggers on headache occurrence or severity is still uncertain and worth investigating with advanced statistical modeling techniques [13, 14].

One of the important methodological issues in studying trigger factors of migraines is the individual models (within-person) and population-level models (using an individual diary) [19]. The impact of trigger factors on migraines was analyzed by using population-level models based on 1099 headache diaries; 30.6% (336/1,099) of the headaches fulfilled the criteria for migraines in this study. A substantial proportion of the reported by migraine sufferers may be a manifestation of mild migraine attacks. These attacks were probable migraines or were treated before the full development of migraine symptoms [36]. There is the possibility of a tension-type headache; the prevalence of tension-type headaches in migraine individuals was similar to the general population [37,38]

The headaches with triggers were more severe and related to migraines in this study. People with triggers frequently had more severe headache profiles. The menstrual migraine is a good example [3, 39–41]. In a study that used paper diaries, hormonal changes demonstrated a hazard ratio increase of up to 96%, while the values for all other factors were less than 35% [42]. Migraineurs with allodynia were reported to have a higher number of triggers relative to those without, indicating that triggers may play a role in the exacerbation of a migraine [43].

Hormonal changes and noise increased the risk of a migraine regardless of preventive medication in this study. Considering that short-term migraine prevention uses triptans for menstrual migraines, conventional preventive medication would not change the features of a headache triggered by menstruation [39]. The associations of stress, overeating, alcohol, and traveling with migraine were not evident with preventive medication. Preventive medication may change the feature of headaches [44].

The study had some limitations. First, the temporal sequence and relationships between the triggers were not evaluated. The changes from the previous levels and associations between the trigger factors may have influenced the headache onset and severity [20]. The differentiation from the premonitory symptoms with functional imaging may be promising [45], Second, we analyzed one-day diaries; the triggers that were present during the preceding 1–3 days were not considered [33, 46], which limits the predictability and direct causality associations between the triggers and headaches. Third, the compliance rate of this study was based on more than 50% of recording period (74.7%), which is not high. However, the recording rate of the SHD (86.3%) observed in this study was comparable to those of previous studies and reasonable for clinical settings [18, 20, 39]. Fourth, most triggers influenced a subgroup of migraineurs, but we did not analyze the risk of migraine by using an individual model [4, 19]. Fifth, we relied on the participants’ judgment for recording the triggers and cannot rule out the possibility of selection bias from the clinical setting and recall or confirmation bias by participant [3].

The merits of this study were analyzing trigger factors in regards to frequency, headache occurrence, headache features, and the influence of preventive medication by using a statistical method and patient-friendly SHD in episodic migraineurs.

## Conclusion

The SHD is an effective tool in the assessment of migraine trigger factors. Headaches with trigger factors had greater severity or migraine features. The type of triggers and the presence of preventive medication may influence headache features, so the investigation of trigger factors is helpful in understanding the pathophysiology of migraines and developing a preemptive strategy for trigger factors.

## Supporting Information

S1 File Dataset of 62 patients, 1099 Headache Diary Records, and 4579 Diary Records. (XLS)

S1 Table Chi-square analysis about the likelihood of Headache and the Number of Triggers. (PDF)
