# Supplementary material for: Analysis of Trigger Factors in Episodic Migraineurs Using a Smartphone Headache Diary Applications
Source: PLoS One. 2016 Feb 22;11(2):e0149577. doi: 10.1371/journal.pone.0149577 (PMC4764678; doi:10.1371/journal.pone.0149577)
Supplement: S1 Table — (PDF) [file pone.0149577.s002.pdf]

**S1 Table. Chi-square analysis about likelihood of Headache and Number of Triggers.**

| Number of Triggers | P-value |
| --- | --- |
| trigger 1 vs 2-9 triggers | 0.95 |
| trigger 1 vs 2 triggers vs 3 vs 4 vs 5-9 | 0.24 |
| trigger 1 vs 2 triggers vs 3-9 triggers | 0.99 |
